# Genome rearrangements and phylogeny reconstruction in *Yersinia pestis*

**DOI:** 10.7717/peerj.4545

**Published:** 2018-03-27

**Authors:** Olga O. Bochkareva, Natalia O. Dranenko, Elena S. Ocheredko, German M. Kanevsky, Yaroslav N. Lozinsky, Vera A. Khalaycheva, Irena I. Artamonova, Mikhail S. Gelfand

**Affiliations:** 1 Kharkevich Institute for Information Transmission Problems, Moscow, Russia; 2 Center for Data-Intensive Biomedicine and Biotechnology, Skolkovo Institute of Science and Technology, Moscow, Russia; 3 Department of Molecular and Chemical Physics, Moscow Institute of Physics and Technology, Moscow, Russia; 4 Department of Bioengineering and Bioinformatics, Lomonosov Moscow State University, Moscow, Russia; 5 Higher Chemical College of the Russian Academy of Sciences, D. Mendeleev University of Chemical Technology of Russia, Moscow, Russia; 6 Stavropol State Agrarian University, Stavropol, Russia; 7 Vavilov Institute of General Genetics Russian Academy of Sciences, Moscow, Russia; 8 Faculty of Computer Science, Higher School of Economics, Moscow, Russia

**Keywords:** Phylogeny reconstruction, Bacteria evolution, Genome rearrangements

## Abstract

Genome rearrangements have played an important role in the evolution of *Yersinia pestis* from its progenitor *Yersinia pseudotuberculosis*. Traditional phylogenetic trees for *Y. pestis* based on sequence comparison have short internal branches and low bootstrap supports as only a small number of nucleotide substitutions have occurred. On the other hand, even a small number of genome rearrangements may resolve topological ambiguities in a phylogenetic tree. We reconstructed phylogenetic trees based on genome rearrangements using several popular approaches such as Maximum likelihood for Gene Order and the Bayesian model of genome rearrangements by inversions. We also reconciled phylogenetic trees for each of the three CRISPR loci to obtain an integrated scenario of the CRISPR cassette evolution. Analysis of contradictions between the obtained evolutionary trees yielded numerous parallel inversions and gain/loss events. Our data indicate that an integrated analysis of sequence-based and inversion-based trees enhances the resolution of phylogenetic reconstruction. In contrast, reconstructions of strain relationships based on solely CRISPR loci may not be reliable, as the history is obscured by large deletions, obliterating the order of spacer gains. Similarly, numerous parallel gene losses preclude reconstruction of phylogeny based on gene content.

## Introduction

*Yersinia pestis*, causing fulminant plague, has evolved clonally from an enteric pathogen, *Yersinia pseudotuberculosis*, that, in contrast, causes a relatively benign enteric illness. Horizontal gene acquisition, massive gene loss, and genome rearrangement events all have played important roles in the evolution of *Y. pestis* from its progenitor (Achtman et al., 1999). *Y. pseudotuberculosis* and *Y. pestis* differ radically in their pathogenesis despite sharing >97% identity in 75% of their chromosomal genes (Martínez-Chavarría & Vadyvaloo, 2015). As only a small number of nucleotide substitutions have occurred, traditional phylogenetic trees of *Y. pestis* strains based on sequence comparison have short internal branches and low bootstraps.

Gene order in prokaryotes is relatively poorly conserved making it a convenient tool for the analysis of the species and strain evolution, when changes in protein, and even gene sequences do not provide sufficient resolution (Wolf et al., 2001). In addition, genome rearrangements are less sensitive to homologous recombination and hence allow for an alternative approach to construction of phylogenetic trees, as even a small number of genome rearrangements may resolve topological ambiguities in a phylogenetic tree (Darling, Miklós & Ragan, 2008).

Among factors affecting genome rearrangement are abundance of mobile elements and the state of repair/recombination systems in the respective genomes (Novichkov et al., 2009). Newly formed pathogens such as *Y. pestis* are known to have a particularly high rate of rearrangements that may be caused by the prevalence of a large variety and number of insert sequences (ISs) (Liang et al., 2014).

The comparison of the *Y. pestis* KIM genome sequence with *Y. pestis* strain CO92 has divided both genomes into 27 conserved segments, and the most parsimonious series of inversions for three multiple-inversion regions has been described (Deng et al., 2002). Further, large-scale genome rearrangements have been described in strains Antiqua, Nepal and Angola (Chain et al., 2006; Eppinger et al., 2010). Comparison of pairs of bacterial genomes has revealed a characteristic “cross-like” pattern of localization of orthologous genes, indicating that inversions around the origin of replication comprise one of the dominant types of genome rearrangements (Eisen et al., 2000).

Multiple genome alignment of nine *Y. pestis* and *Y. pseudotuberculosis* genomes has featured universal Locally Collinear Blocks (LCBs) yielding seven parsimonious scenarios of the inversion history. The reconstructed pattern of genome rearrangements confirms strong preference for the replichore balance and over-representation of “symmetric inversions”—inversions with endpoints that are equally distant from the origin of chromosomal replication (Darling, Miklós & Ragan, 2008).

Several algorithms based on a variety of optimization approaches have been developed for the reconstruction of the rearrangement history (Avdeyev et al., 2016; Hu, Lin & Tang, 2014). However, reconstruction for large datasets remains a challenge, since the minimum length series of inversions (the optimal sorting path) is often not unique and equally many optimal sorting paths exist (Miklós & Darling, 2009).

Later, the LCB model has been used to infer the phylogenetic relationships among eight complete *Y. pestis* genomes from the breakpoint distance matrix, yielding the conclusion that the pattern of *Y. pestis* chromosome rearrangements reflects the genetic features of specific geographical areas and might be applied to distinguish *Y. pestis* isolates (Liang et al., 2010). A set of gene families from thirteen *Yersinia* species has been used to reconstruct a complete genome sequence for the ancestor, integrating information from the sequences, the species tree, and the gene order (Duchemin, Daubin & Tannier, 2015).

Being a traditional object for the spoligotyping, a special type of genotyping based on the spacer nucleotide analysis, CRISPR systems of *Y. pestis* strains often serve as a model for CRISPR-based evolutionary studies. All three separate genomic CRISPR loci have been described in detail (Pourcel, Salvignol & Vergnaud, 2005), including numerous strains without complete genomes (Vergnaud et al., 2007; Cui et al., 2008; Riehm et al., 2012; Barros et al., 2014; Riehm et al., 2015). Relationships between strains have been studied using the distance based on shared and differential spacers content only (Barros et al., 2014) or taking into account the principles of evolutionary cassette dynamics. In particular, the evolutionary history of *Y. pestis* based on CRISPR polymorphism has been reconstructed in the form of an acyclic oriented graph (Cui et al., 2008). Later, a general mathematical model of CRISPR evolution has been applied to reconstruct the relationships of strains for each of the three CRISPR loci (Kupczok & Bollback, 2013).

Here, we integrate the history at different levels of genome evolution, including gene flux, sequence divergence, chromosome segmental inversions, and spacer acquisitions and deletions in CRISPR cassettes, for genomes of twelve completely sequenced *Y. pestis* strains and four *Y. pseudotuberculosis* strains.

## Materials and Methods

### Genomes

Complete genome sequences of four *Yersinia pseudotuberculosis* and twelve *Yersinia pestis*, all available as of August 1st, 2013, were taken from the NCBI Genome database (Benson et al., 2015) and are listed in Table S1.

### Construction of orthologs

Bidirectional best hits (BBHs) were constructed for each pair of strains using BLASTP (Zhang & Madden, 1997). BLASTP hits with identity <50% or coverage of the shorter sequence <67% were ignored. At the next step, if paralogs were more similar to each other than to either BBH partner, both paralogs were added to the orthologous group. Then, maximal connected components were constructed. This was done using *ad hoc* software based on the Relational Database Management System (RDBMS) Oracle Database Express Edition.

### Trees based on nucleotide alignments

First we performed codon alignment and filtering for each of the 2117 orthologous groups using Mafft version v7.123b (Katoh & Standley, 2013) and Guidance 2.01 (Penn et al., 2010). Orthologous groups containing sequences with score below 0.8 were excluded from further analysis. Poorly aligned residues (guidance score below 0.8) were masked. The resulting sequences were concatenated and the tree was constructed with RAxML v8.2.9 (Stamatakis, 2014) using the GTR+Gamma model with 100 bootstrap runs.

### Synteny blocks reconstruction

Synteny blocks were constructed using the Sibelia algorithm (Minkin et al., 2013) with the block length threshold 5,000 bp. To ensure robustness of the tree topology relative to this parameter we also performed calculations with the block length thresholds of 500 bp and 2,000 bp (see  Figs. S2– S4). We used two approaches for the blocks construction. Splitting the chromosomes on non-repetitive common blocks was used for inversions analysis as the construction of all types of blocks allowed us to consider all type of rearrangements such as losses and gains.

### Trees based on gene order

Trees based on gene order were build using two approaches. The trees using the Maximum Likelihood approach for the gene order were constructed using the MLGO software (Hu, Lin & Tang, 2014) for two data sets, synteny blocks having only one copy in every genome, and all synteny blocks found in these genomes. For both datasets we performed 1,000 replicates for the bootstrap analysis. The phylogenetic network was obtained using the Bayesian model of genome rearrangements by inversions implemented in the BADGER software (Larget, Simon & Kadane, 2002). We calculated 1,510,000 modification proposal steps, discarded the first 10,000 steps of each chain as burn-in and then subsampled every 50 steps as described in (Darling, Miklós & Ragan, 2008). The convergence of the Markov chain was assessed across multiple independent runs of BADGER as recommended in the BADGER manual. We used SplitsTree v4 (Huson & Bryant, 2006) for the network visualization.

### Trees based on CRISPR cassettes composition

CRISPR cassettes were downloaded from CRISPRdb (Grissa, Vergnaud & Pourcel, 2007). Phylogenetic trees were reconstructed manually based on the CRISPR cassettes evolution rules. At that, two types of events were allowed, addition of a new spacer at the leader end, and deletion of one or several adjacent spacers from any part of a cassette. We further assumed (1) no independent additions of the same spacer to two different cassettes; (2) rare, but possible independent deletions of the same cassette segments; and (3) more probable single deletion of a segment including several adjacent spacers compared to several subsequent deletions of the segment parts.

**Figure 1:**
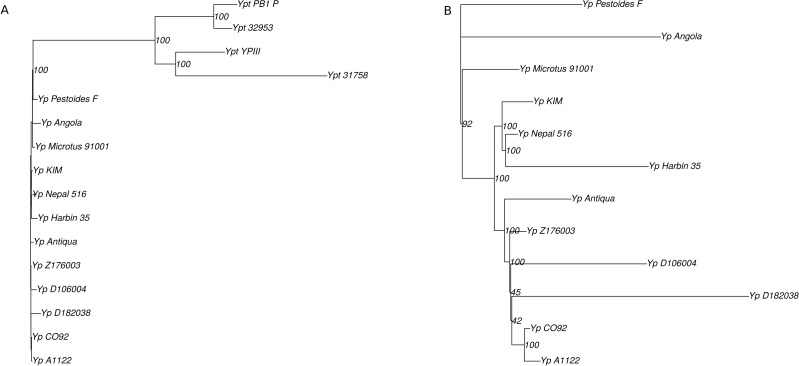
(A) Phylogenetic tree of the *Yersinia* spp., based on nucleotide alignments of 2408 single-copy universal genes; (B) phylogenetic tree of the *Y. pestis* branch only.

## Results and Discussion

### Phylogenetic trees based on sequences alignments

The phylogenetic tree for the analyzed *Y. pseudotuberculosis* and *Y. pestis* was constructed based on 2408 single-copy universal genes using a concatenation of individual nucleotide alignments (Fig. 1). We used the *Y. enterocolitica* genome to root the tree. We observed that *Y. pestis* strains formed a clade within the *Y. pseudotuberculosis* subtree, in agreement with previous genome analyses (Chain et al., 2006; Rasmussen et al., 2015).

There seemed to be several key noise factors. A small number of nucleotide substitutions resulted in low bootstrap values in several vertices, e.g., for Z176003, CO92, and A1122. Also, homologous recombination events might dramatically influence the tree topology reconstruction and lead to low level of bootstrap supports.

### Phylogenetic trees based on gene order

**Figure 2:**
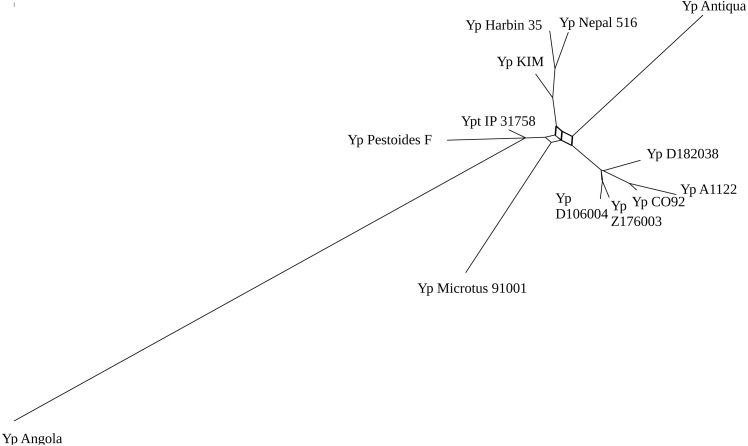
Phylogenetic trees network of the *Yersinia pestis* with Bayesian posterior probability threshold = 0.1.

As genome rearrangements had played an important role in the evolution of *Yersinia pestis*, we constructed phylogenetic trees based on the gene order to check the topology and resolve nodes with low-level bootstrap support. Based on whole-genome alignments, 123 synteny blocks that were common for all strains under consideration with length more than 5,000 bp were obtained. We applied the Bayesian model of genome rearrangements by inversions and visualized phylogenetic tree signal as a consensus network (Fig. 2). This network has a complicated structure with ambiguous positions of the long branches but it has well-resolved clades with closely related strains that are not resolved in the alignment-based tree (Fig. 1B).

**Figure 3:**
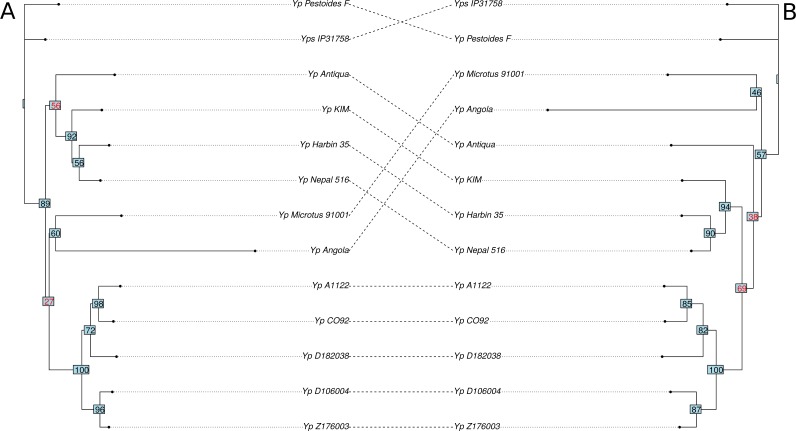
Phylogenetic trees of the *Yersinia* spp. based on gene order. (A) Optimal topology based on inversions. (B) Optimal topology based on all types of rearrangements. Nodes that produce differences in the trees are labeled in red.

Application of the Maximum Likelihood approach (Hu, Lin & Tang, 2014) to synteny blocks common for all *Y. pestis* strains revealed the optimal tree topology (Fig. 3A). Strains D106004 and Z176003 formed a separate branch in the inversions-based tree due to the same inversion with length about 150 kB that had occurred in these strains and D182038 that was an outgroup; at that, in the sequence-based tree Z176003 was an outgroup with a low bootstrap support of this node (Fig. 1B). One more parallel inversion with length about 350 kB was found in A1122 and D182038 that had lead to a low support in this node in the inversions-based tree. The boundaries of the inversions are formed by repeated sequences (transposases).

**Figure 4:**
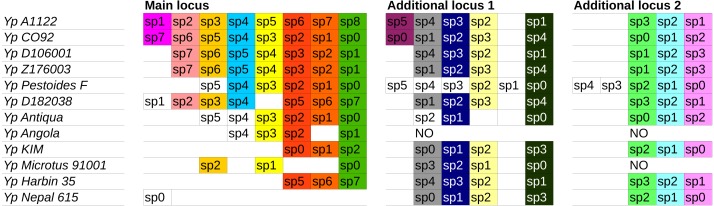
CRISPR cassettes of completely sequenced *Y. pestis* strains. Cassette IDs and spacer numbers are given according to CRISPRdb (Grissa, Vergnaud & Pourcel, 2007). Identical spacers are shown by the same color; unique spacers are set in frames.

The observed parallel inversions could be explained by homologous recombination (horizontal transfer between strains) involving a segment containing the inverted fragments. If this were the case, sequence trees constructed using the genes from the inverted fragments would cluster together strains with the parallel inversions. However, the trees for both inversions are poorly resolved and hence provide no information about possible horizontal transfer ( Fig. S1).

One more short parallel inversion was found in *Y. pestis* KIM and *Y. pestis* Nepal at decreased synteny length threshold ( Fig. S4). The inverted block of length 3,500 bp contained integrase, antibiotic biosynthesis monooxygenase, dihydroorotase, DNA damage-inducible protein I, biofilm formation regulatory protein BssS, and IS256 family transposase. A possible explanation could be an incorporation of a mobile element in different orientations.

Adding the information about non-common blocks leads to decrease of the bootstrap supports (Fig. 3B). This may be explained by numerous parallel gains and losses natural to fast-evolving bacterial genomes such as recently formed pathogens. In particular, the Antiqua strain moves to the Microtus node, in agreement with the fact that, according to the ability to ferment glycerol and to reduce nitrate, strains Antiqua, Pestoides, Microtus, and Angola belong to the Antiqua biovar (Chain et al., 2006).

Based on the phylogenetic patterns, most events are losses, with only three blocks likely to have been inserted and three blocks having mosaic patterns that cannot be interpreted. However, as the latter have the same position in the genomes, they probably represent parallel losses. The inserted blocks are a prophage insertion, a fragment with a gene encoding a penicillin-binding protein and a transposase, and a gene encoding domains of an invasin-like inverse autotransporter protein.

**Figure 5:**
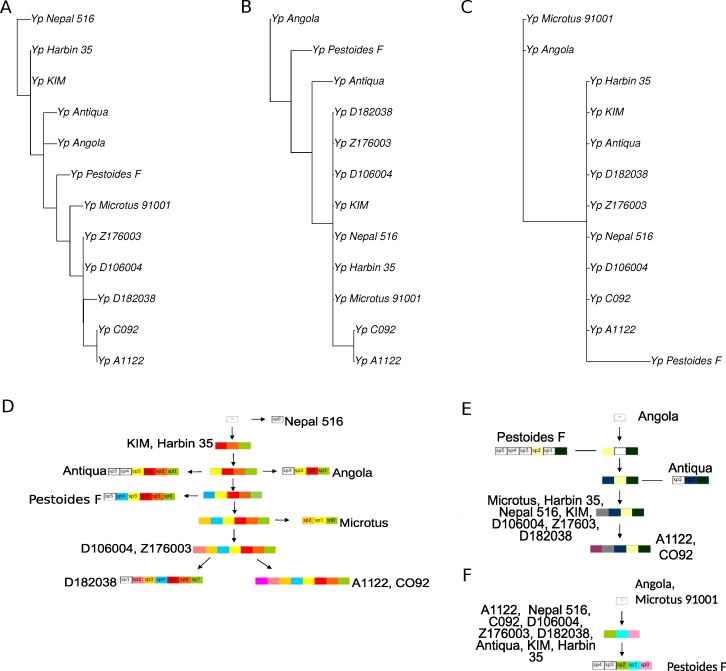
Cladograms (A, B, C) and schemas of evolution (D, E, F) of three CRISPR loci of *Y. pestis*. (A, D) The main, most variable, locus; (B, E) additional locus 1; (C, D) additional locus 2.

**Figure 6:**
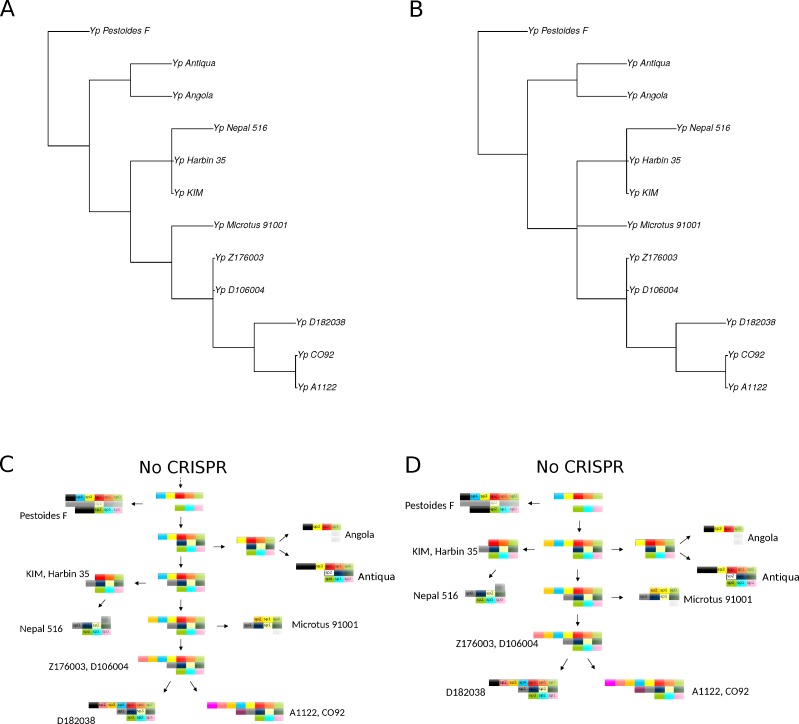
Cladograms (A, B) and schemas of evolution (C, D) of two integrated CRISPR-based maximum parsimony phylogenetic trees most compatible with the sequence tree.

### CRISPR analysis

CRISPR cassettes of the considered *Y. pestis* strains are shown in Fig. 4. Initially, we constructed separate phylogenetic trees for each of the three CRISPR loci using the parsimony approach (Fig. 5, see ‘Methods’). As the number of events in each locus was small, the history of each locus could be reconstructed unambiguously.

However, the genome of *Y. pestis* evolves as a whole and the individual histories of the loci should be reconciled. In this case the reconstruction is ambiguous, as there are two equivalent reconstructions of the common ancestors and five equal positions of the Nepal strain on the maximum parsimony tree. Two maximum parsimony trees most compatible with the sequence tree are shown in Fig. 6. The trees constructed based on nucleotide sequences or rearrangements satisfy the rules of CRISPR cassette evolution (see Methods), but each of them implies two additional losses of cassette segments in comparison with the maximum parsimony tree. In particular, the sequence-based tree implies two independent parallel losses of the same segments of the main locus in the Angola and Antiqua strains branches.

No direct evidence for homologous recombination or horizontal transfer of complete CRISPR loci or smaller groups of spacers was observed. While the parallel losses could be interpreted as a sign of homologous recombination/horizontal transfer, parallel events seem more likely, given the overall high rate of spacer loss. Generally, the problem of horizontal transfer vs. parallel events, duplications, and losses is a difficult one in the comparative genomics of prokaryotes (Koonin, 2016).

## Conclusions

Detailed reconstruction of evolution of bacterial strains provides a framework for epidemiological studies and analysis of acquired pathogenesis loci and drug resistance determinants.

Using *Y. pestis* as an example, we demonstrate that integrated analysis of sequence-based and inversion-based trees enhances the resolution of the phylogenetic reconstruction. At that, inversions may resolve branches with low bootstrap support.

In contrast, reconstructions of strain relationships based on solely CRISPR loci may not be reliable, as the history is greatly obscured by large deletions, obliterating the order of spacer gains. Even less reliable seem to be reconstructions based on shared spacer content. Similarly, numerous parallel gene losses preclude reconstruction of phylogeny based on gene content.

## Supplemental Information

10.7717/peerj.4545/supp-1 Figure S1 Phylogenetic trees for *Y. pestis* genes involved in the parallel inversions (A) in A1122 and D182038 and (B) in Z176003 and D106004.

10.7717/peerj.4545/supp-2 Figure S2 Phylogenetic trees network of the *Y. pestis* Calculations with the block length thresholds of 500 bp.

10.7717/peerj.4545/supp-3 Figure S3 Phylogenetic trees of the *Y. pestis* based on gene order Calculations with the block length thresholds of 500 bp. (A) Optimal topology based on inversions; (B) Optimal topology based on all types of rearrangements.

10.7717/peerj.4545/supp-4 Figure S4 Phylogenetic trees of the *Y. pestis* based on gene order Calculations with the block length thresholds of 2,000 bp. (A) Optimal topology based on inversions; (B) Optimal topology based on all types of rearrangements.

10.7717/peerj.4545/supp-5 Table S1 Strains under consideration

## Additional Information and Declarations

### Data Availability

The following information was supplied regarding data availability: Github: https://github.com/OlgaBochkaryova/yersinia-phylogeny-analysis.git.
