# Supplementary figures and images for: Genome rearrangements and phylogeny reconstruction in Yersinia pestis
Source: PeerJ. 2018 Mar 27;6:e4545. doi: 10.7717/peerj.4545 (PMC5877447; doi:10.7717/peerj.4545)

**A**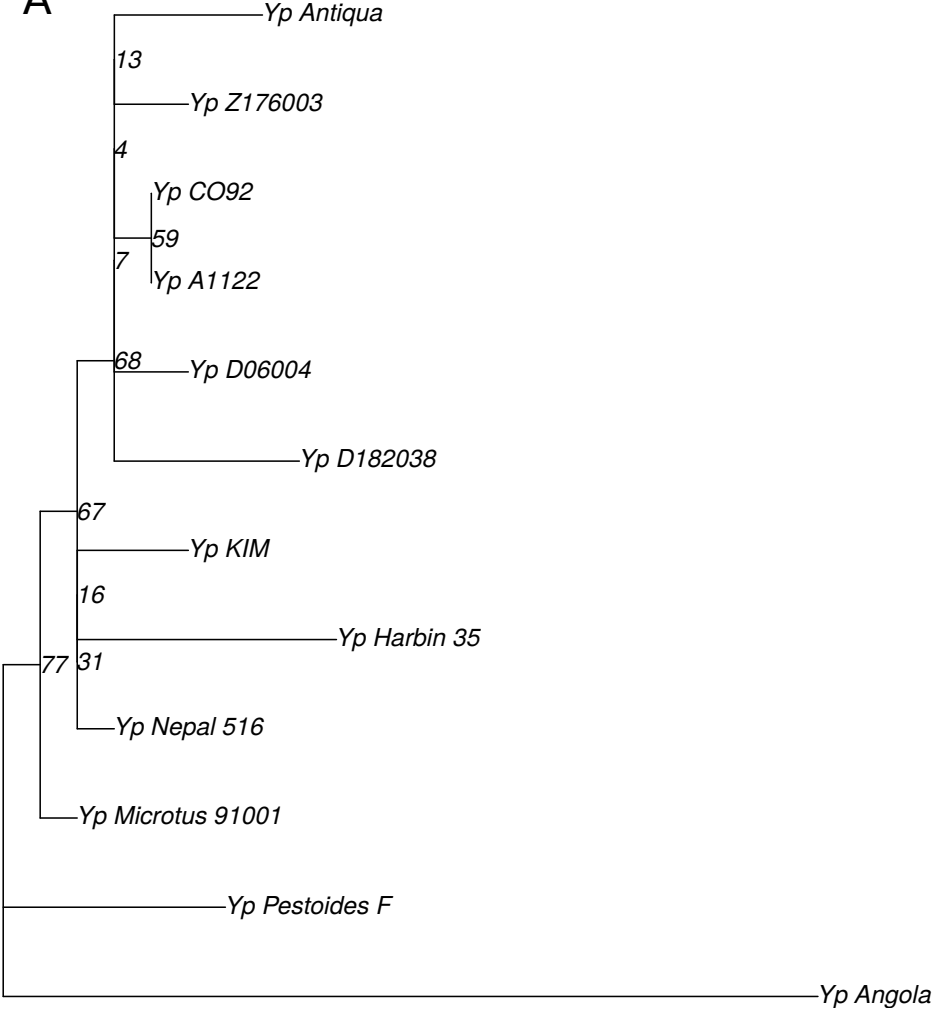**B**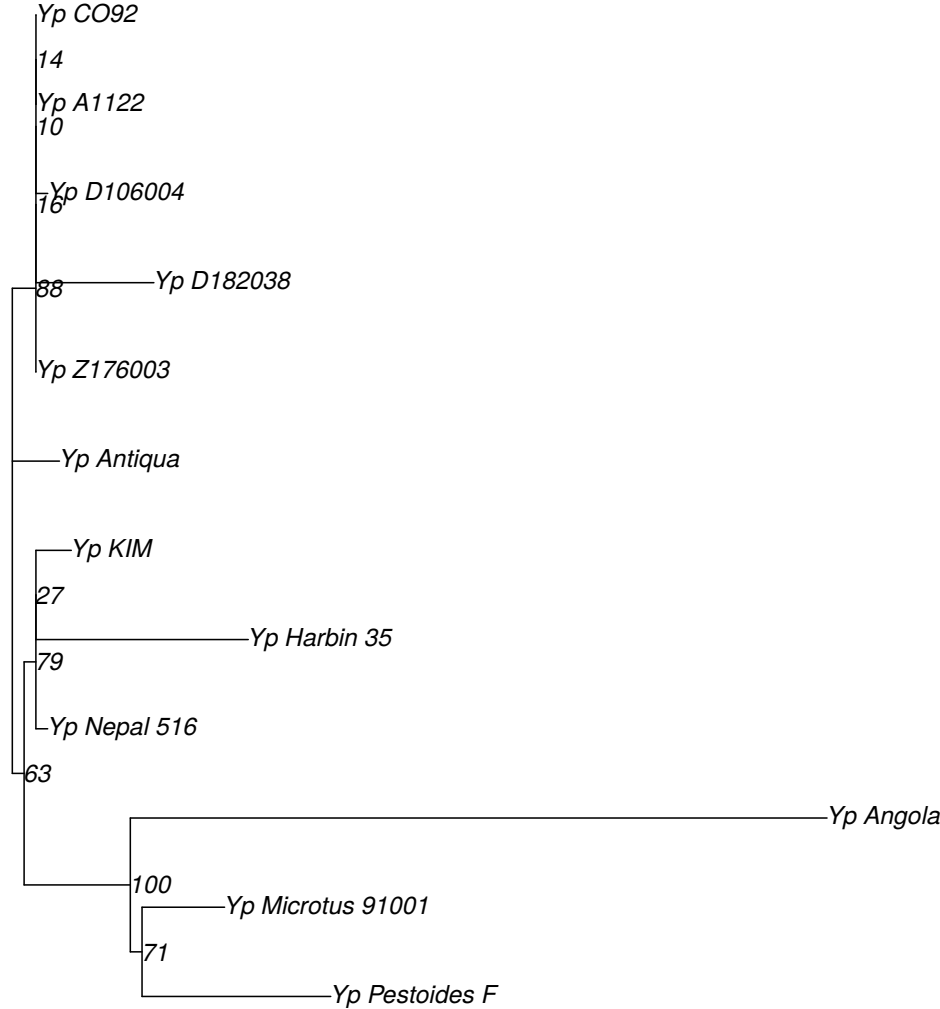

Supplement: Figure S1 — (A) in A1122 and D182038 and (B) in Z176003 and D106004. [file peerj-06-4545-s001.pdf]

10.0

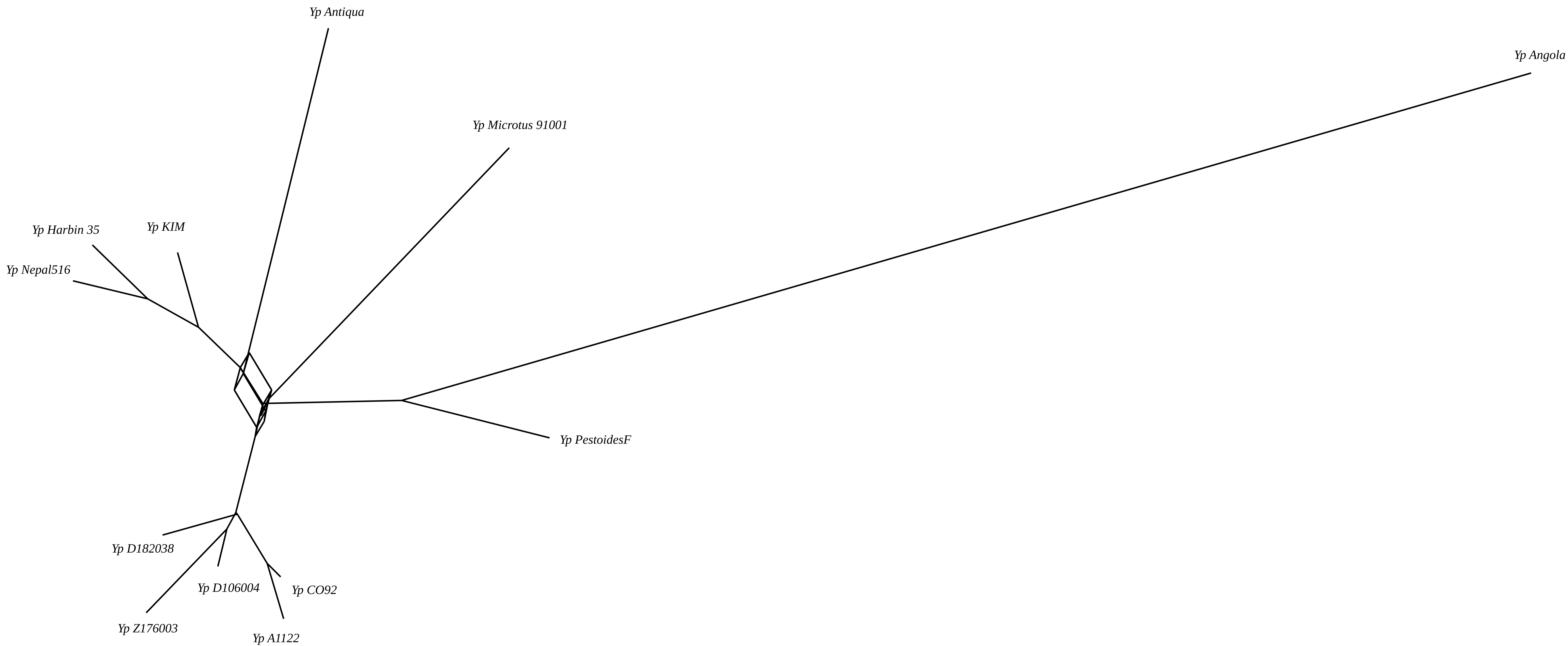

Supplement: Figure S2 — Calculations with the block length thresholds of 500 bp. [file peerj-06-4545-s002.pdf]

# A

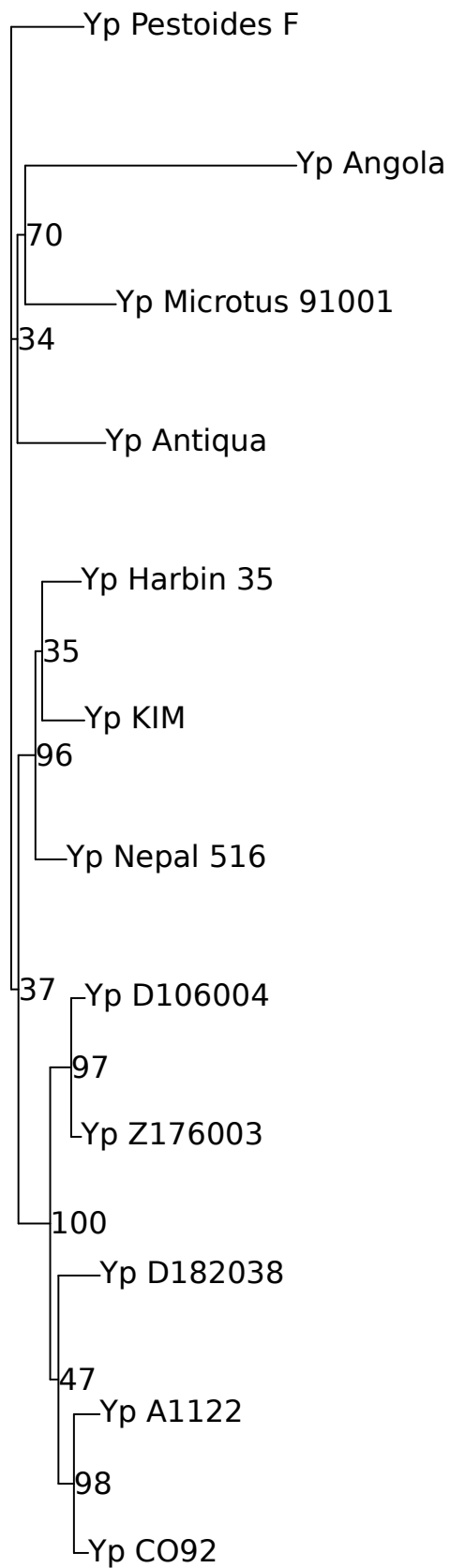

# B

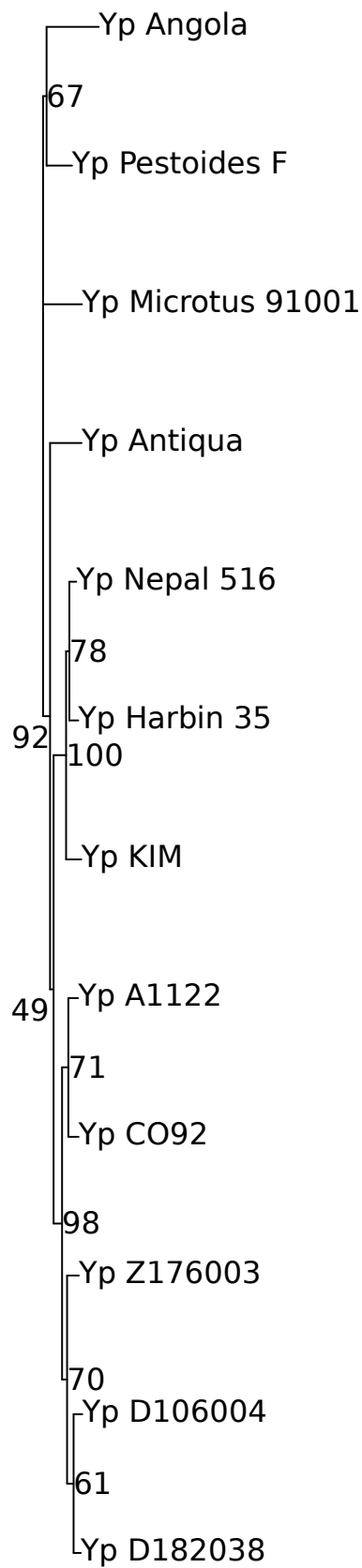

Supplement: Figure S3 — Calculations with the block length thresholds of 500 bp. (A) Optimal topology based on inversions; (B) Optimal topology based on all types of rearrangements. [file peerj-06-4545-s003.pdf]

A

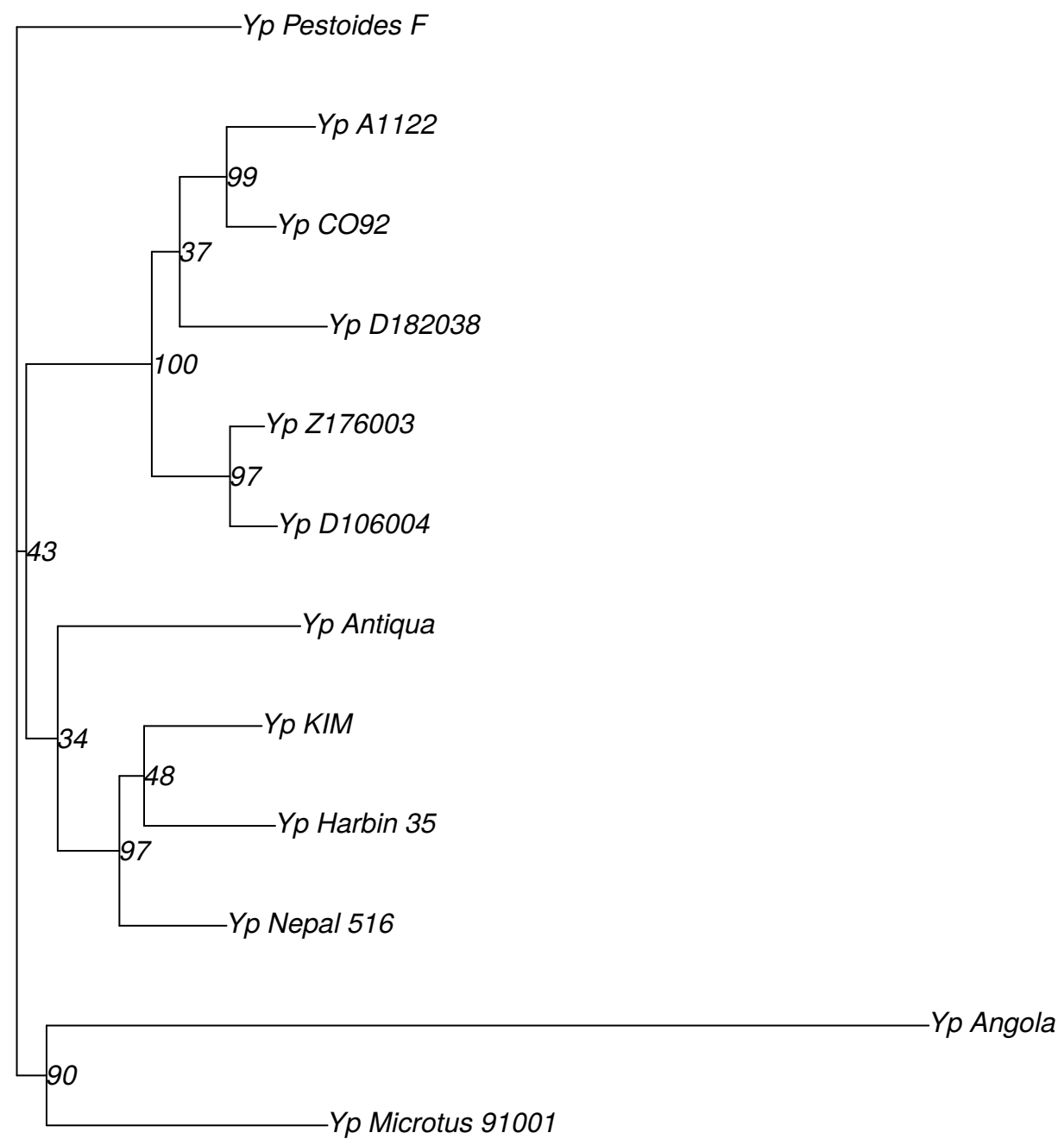

B

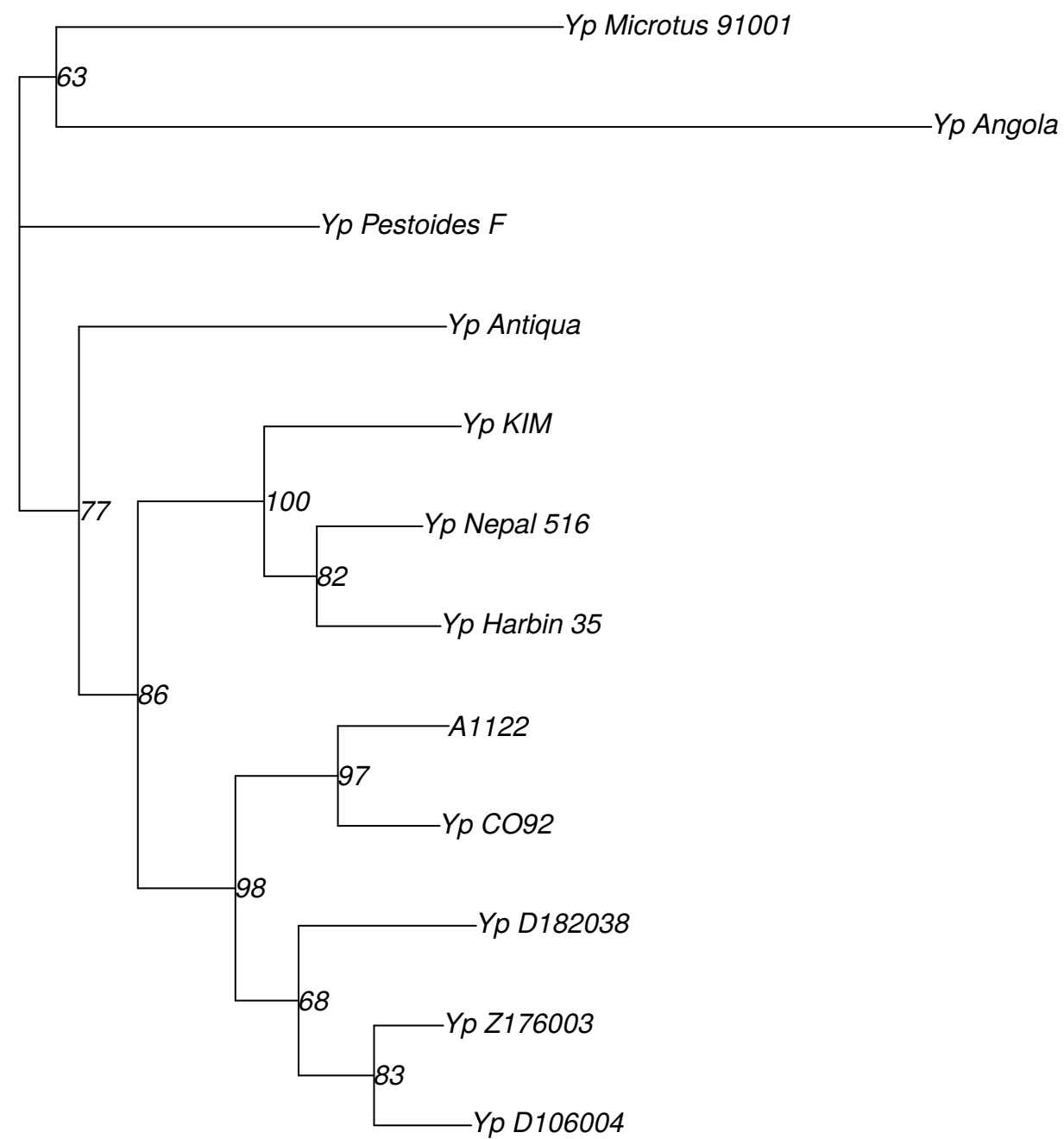

Supplement: Figure S4 — Calculations with the block length thresholds of 2,000 bp. (A) Optimal topology based on inversions; (B) Optimal topology based on all types of rearrangements. [file peerj-06-4545-s004.pdf]
