# Supplementary material for: Genome rearrangements and phylogeny reconstruction in Yersinia pestis
Source: PeerJ. 2018 Mar 27;6:e4545. doi: 10.7717/peerj.4545 (PMC5877447; doi:10.7717/peerj.4545)
Supplement: Table S1 [file peerj-06-4545-s005.pdf]

Table S1. Strains under consideration.

N	Strain	NCBI Sequence	Short name
1	*Yersinia enterocolitica* subsp. *palearctica* 105.5R(r)	NC_015224	outgroup
2	*Yersinia pestis* A1122	NC_017168	*Yp* A1122
3	*Yersinia pestis* Angola	NC_010159	*Yp* Angola
4	*Yersinia pestis* Antiqua	NC_008150	*Yp* Antiqua
5	*Yersinia pestis* biovar Medievalis str. Harbin 35	NC_017265	*Yp* Harbin 35
6	*Yersinia pestis* biovar Microtus str. 91001	NC_005810	*Yp* Microtus 91001
7	*Yersinia pestis* D106004	NC_017154	*Yp* D106004
8	*Yersinia pestis* D182038	NC_017160	*Yp* D182038
9	*Yersinia pestis* KIM10+	NC_004088	*Yp* KIM
10	*Yersinia pestis* Nepal516	NC_008149	*Yp* Nepal 516
11	*Yersinia pestis* Pestoides F	NC_009381	*Yp* Pestoides F
12	*Yersinia pestis* Z176003	NC_014029	*Yp* Z176003
13	*Yersinia pestis* CO92	NC_003143	*Yp* CO92
14	*Yersinia pseudotuberculosis* IP 31758	NC_009708	*Ypt* IP31758
15	*Yersinia pseudotuberculosis* IP32953	NC_006155	*Ypt* IP32953
16	*Yersinia pseudotuberculosis* PB1/+	NC_010634	*Ypt* PB1
17	*Yersinia pseudotuberculosis* YPIII	NC_010465	*Ypt*YPIII
